# Social comparison, personal relative deprivation, and materialism

**DOI:** 10.1111/bjso.12176

**Published:** 2016-11-23

**Authors:** Hyunji Kim, Mitchell J. Callan, Ana I. Gheorghiu, William J. Matthews

**Affiliations:** ^1^ Department of PsychologyUniversity of EssexColchesterUK; ^2^ Department of PsychologyUniversity of CambridgeUK

**Keywords:** social comparison, personal relative deprivation, materialism, consumer behaviour, social status

## Abstract

Across five studies, we found consistent evidence for the idea that personal relative deprivation (PRD), which refers to resentment stemming from the belief that one is deprived of deserved outcomes compared to others, uniquely contributes to materialism. In Study 1, self‐reports of PRD positively predicted materialistic values over and above socioeconomic status, personal power, self‐esteem, and emotional uncertainty. The experience of PRD starts with social comparison, and Studies 2 and 3 found that PRD mediated the positive relation between a tendency to make social comparisons of abilities and materialism. In Study 4, participants who learned that they had less (vs. similar) discretionary income than people like them reported a stronger desire for more money relative to donating more to charity. In Study 5, during a windfall‐spending task, participants higher in PRD spent more on things they wanted relative to other spending categories (e.g., paying off debts).

## Background

Materialism is generally defined as ‘individual differences in people's endorsement of values, goals, and associated beliefs that center on the importance of acquiring money and possessions that convey status’ (Dittmar, Bond, Hurst, & Kasser, 2014, p. 880). Understanding the psychological factors that correlate with materialism is important because of its potential negative implications for individuals and for society (Kasser, 2002). For example, people higher in materialism tend to have lower personal well‐being (Dittmar *et al*., 2014) and greater financial debt (Garðarsdóttir & Dittmar, 2012), and are less concerned about environmental issues (Hurst, Dittmar, Bond, & Kasser, 2013).

Given these potential negative consequences, researchers have been interested in understanding the antecedents of materialism (see, e.g., Kasser, Ryan, Couchman, & Sheldon, 2004; Shrum *et al*., 2014). Experimental and correlational research has shown, for example, that low self‐esteem (Park & John, 2011), low sense of personal power (Rucker & Galinsky, 2008), feelings of uncertainty (Chang & Arkin, 2002), economic threat (Sheldon & Kasser, 2008), perceived peer pressure (Banerjee & Dittmar, 2008), and lower income (Kasser, Ryan, Zax, & Sameroff, 1995) contribute to people adopting materialistic values and goals. Cutting across these explanations is the idea that increased materialism is ‘one type of compensatory strategy intended to countermand the distressing effects of feelings of insecurity’ (Kasser *et al*., 2004, p. 13; see also Shrum *et al*., 2014). Put differently, according to these accounts, some people place greater importance on money, possessions, and status because they believe having these things will, in some way, reduce their feelings of insecurity, increase their sense of personal power, or improve their self‐worth.

In the current research, we examined whether personal relative deprivation (PRD) also relates to materialism. Personal relative deprivation refers to dissatisfaction and resentment resulting from the belief that one is deprived of desired and deserved outcomes compared with what others have (for reviews, see Crosby, 1976; Smith & Pettigrew, 2014; Smith, Pettigrew, Pippin, & Bialosiewicz, 2012). According to Smith *et al*. (2012), the experience of PRD stems from a process whereby an individual makes a social comparison on a given outcome, believes themselves to be unfairly disadvantaged, and consequently feels resentful and dissatisfied. One key aspect of this process is that *comparative* judgements of one's status – and not simply one's objective or absolute status – can elicit a sense of unfairness and resentment. For example, learning that a work colleague has similar outputs but a higher salary than you can elicit a sense of unfairness and resentment, even though you might not be ‘objectively’ deprived in terms of your absolute income. Thus, even people who are ‘well off’ can feel resentful about their lot in life, whereas those with minimal financial resources may not necessarily feel unfairly disadvantaged (Crosby, 1976).

Some research has shown that lower socioeconomic status is associated with higher materialism (e.g., Chang & Arkin, 2002; Kasser *et al*., 1995; Zhang, Howell, & Howell, 2016), presumably because, as Kasser *et al*. (2004) suggested, some people who are socioeconomically disadvantaged might compensate for their feelings of inadequacy or insecurity by adopting materialistic values and goals. However, people who feel *relatively* deprived, regardless of their absolute material standing, may also place importance on acquiring money and possessions to compensate for the sense that they are getting less than they deserve relative to others. That is, resentment stemming from unfavourable social comparisons might lead some people to orient towards materialism, even if they are otherwise ‘objectively’ affluent. This analysis fits well with research showing that, because believing oneself to be worse off than similar others is aversive (Callan, Kim, & Matthews, 2015a), people often adopt various strategies to minimize feeling relatively deprived (e.g., by gambling, or by improving one's professional qualifications; see Smith *et al*., 2012, for a review). We investigated the adoption of materialistic values and goals as one such strategy.

### Overview of current research

In five studies, we examined whether people higher in PRD place greater importance on materialistic values and goals. As Dittmar *et al*. (2014) highlighted in their recent meta‐analysis, two broad types of measures of materialism exist in the literature: (1) those that ask respondents to rate their agreement with statements concerning materialistic beliefs, behaviours, and values (e.g., the Material Values Scale [MVS]; Richins & Dawson, 1992); and (2) those where participants indicate the importance they place on goals for financial and material success (e.g., the Aspiration Index; Kasser & Ryan, 1993). We employed both of these types of measures. Study 1 examined whether individual differences in PRD are associated with the endorsement of material values over and above several individual difference factors that might confound this relationship.

Personal relative deprivation requires social comparison. In Studies 2 and 3, we examined whether a tendency to engage in social comparison correlates with the perceived importance of financial success (Study 2) and material values (Study 3) through PRD. In Study 4, we sought causal evidence for the idea that PRD contributes to materialism by experimentally manipulating adverse social comparisons and assessing the degree to which participants placed importance on acquiring more income relative to giving more to charity. In Study 5, we examined whether participants higher in PRD would report spending more money on things they wanted relative to other types of expenditures (e.g., donating to charity, investments) during an imaginary windfall‐spending task.

### Participant sampling

Across our studies, the minimum required sample sizes were based on the sample size required to obtain 80% power (usually much higher) to detect ‘medium’ effect sizes (e.g., *d *=* *0.45 for Study 4) at *p *<* *.05 (two‐tailed). However, the final sample sizes were not completely predetermined because of the unpredictable nature of participant recruitment (e.g., excessive sign‐ups, removal of participants who failed an attention check).

## STUDY 1

Consistent with the preceding theoretical analysis, Zhang, Tian, Lei, Yu, and Liu (2015) found that self‐reported PRD correlated positively with endorsement of material values. One issue that has yet to be resolved, however, is whether PRD uniquely contributes to materialism over and above relevant individual difference factors that might confound their relationship. That is, people higher in PRD might endorse material values simply because they also feel less powerful and more uncertain, have lower self‐esteem, or are lower in SES (cf. Kasser *et al*., 2004). In Study 1, we aimed to establish whether individual differences in PRD correlate positively with materialism over and above these factors.

## Method

### Participants

Participants from the United States (*N *=* *393; 230 males; *M*_age_ = 35.37, *SD*_age_ = 11.25) were recruited through Amazon's Mechanical Turk. Eight additional participants were excluded because of duplicate IP addresses.

### Procedure and measures

Participants completed the measures listed below. The first four measures listed below were presented in random order between participants. None of the measures included an attention check item.

#### Personal relative deprivation

We used Callan, Shead, and Olson's (2011) five‐item Personal Relative Deprivation Scale (PRDS) to measure individual differences in PRD. The PRDS measures people's general beliefs and feelings associated with comparing their outcomes to the outcomes of similar others (e.g., ‘I feel deprived when I think about what I have compared to what other people like me have’; ‘I feel privileged compared to other people like me’). The PRDS asks participants to make their ratings of what they have relative to *similar others* because such comparisons (vs. comparisons with dissimilar others) provide the most diagnostic information for self‐evaluation (Callan *et al*., 2015a; Festinger, 1954; Wood, 1989).

The PRDS has acceptable test–retest and internal reliability (Callan *et al*., 2015a) and predicts theoretically relevant consequences of higher PRD, including lower self‐esteem (Callan, Ellard, Shead, & Hodgins, 2008), increased gambling urges among gamblers (Callan, Shead, & Olson, 2015), delay discounting (Callan *et al*., 2011; Mishra & Novakowski, 2016), and poorer self‐rated health (Callan *et al*., 2015a; Mishra & Carleton, 2015). Participants responded to the items using a 6‐point scale (1 = *strongly disagree*, 6 = *strongly agree*). Higher values indicate higher PRD.

#### Personal sense of power

We measured personal sense of power using Anderson, John, and Keltner's (2012) eight‐item Personal Sense of Power Scale (e.g., ‘In my relationship with others, I can get people to listen to what I say’; ‘I think I have a great deal of power’). Participants rated the items using a 7‐point scale (1 = *Disagree strongly*, 7 = *Agree strongly*). Higher values indicate higher personal sense of power.

#### Self‐esteem

We assessed self‐esteem using Rosenberg's (1965) Self‐Esteem Scale (e.g., ‘On the whole, I am satisfied with myself’; ‘At times I think I am no good at all’). Participants rated the items using a 4‐point scale (1 = *Strongly agree*, 4 = *Strongly disagree*); higher values indicate higher self‐esteem.

#### Emotional uncertainty

We employed the 15‐item emotional uncertainty subscale of the Uncertainty Response Scale (Greco & Roger, 2001; e.g., ‘I feel anxious when things are changing’; ‘I get worried when a situation is uncertain’). Participants indicated how often they experienced each statement using a 4‐point scale (1 = *Never*, 4 = *Always*). Higher values indicate greater emotional uncertainty.

#### Materialism

To assess materialistic values, we used Richins and Dawson's (1992) widely used 18‐item MVS (e.g., ‘I admire people who own expensive homes, cars, and clothes’; ‘The things I own aren't all that important to me’; ‘It sometimes bothers me quite a bit that I can't afford to buy all the things I'd like’). Participants rated the item using a 5‐point scale (1 = *Strongly disagree*, 5 = *Strongly agree*), and higher values indicate greater materialism.

#### Income and education

Participants reported their annual household income before taxes by selecting one of eight categories (1 = *less than $15,000*, 2 = *$15,001 to $25,000*, 3 = *$25,001 to $35,000*, 4 = *$35,001 to $50,000*, 5 = *$50,001 to $75,000*, 6 = *$75,001 to $100,000*, 7 = *$100,001 to £150,000*, 8 = *greater than $150,000*). Across our studies, we coded income responses using the category mid‐points, with the value for the open‐ended top category being Parker and Fenwick's (1983) median‐based estimator. Participants also indicated their highest level of educational attainment (1 = *did not finish high school*, 2 = *high school graduation*, 3 = *college graduation*, 4 = *postgraduate degree*), which we treated as a continuous variable following previous research (e.g., Kraus, Adler, & Chen, 2013).

## Results and discussion

Descriptive statistics, alpha reliabilities, and correlations among the measures are shown in Table 1. Personal relative deprivation, sense of power, self‐esteem, and emotional uncertainty all correlated significantly with materialism in the expected directions. Neither income nor educational attainment correlated significantly with materialism. Crucially, a multiple regression analysis showed that PRD accounted for significant incremental variance in materialism over and above sense of power, self‐esteem, emotional uncertainty, annual income, and education attainment (see Table 2). Therefore, the relation between PRD and materialism does not appear to be confounded by these individual difference factors that have been shown previously to contribute to material values.

**Table 1:** Descriptive statistics and intercorrelations for measures used in Study 1

Measures	*M* (*SD*)	1.	2.	3.	4.	5.	6.	7.
1. PRDS	3.20 (1.15)	(.87)						
2. Power	4.69 (1.24)	−.37^a^	(.93)					
3. Self‐esteem	2.98 (.64)	−.47^a^	.48^a^	(.94)				
4. Uncertainty	2.11 (.64)	.47^a^	−.41^a^	−.49^a^	(.94)			
5. Materialism	2.76 (.72)	.49^a^	−.12^a^	−.29^a^	.33^a^	(.91)		
6. Income ($)	52.4k (38.2k)	−.32^a^	.20^a^	.18^a^	−.16^a^	.03	–	
7. Education	2.74 (.66)	−.19^a^	.14^a^	.15^a^	−.21^a^	−.07	.34^a^	–

Note

PRDS = Personal Relative Deprivation Scale; Power = Personal Sense of Power Scale.

When applicable, alpha reliabilities are presented in parentheses along the diagonal.

a *p *<* *.05.

**Table 2:** Multiple regression analysis for Study 1

Predictors	Material values			
	*b* (*SE*)	95% CI for *b*	β	*sr* ^2^
PRDS	0.31 (0.03)	0.24, 0.37	.49^a^	.16
Power	0.06 (0.03)	0.01, 0.12	.11^a^	.009
Self‐esteem	−0.08 (0.06)	−0.20, 0.04	−.07	.003
Uncertainty	0.16 (0.06)	0.04, 0.28	.14^a^	.01
Income	4e‐6 (9e‐7)	2e‐6, 6e‐6	.21^a^	.04
Education	−0.02 (0.05)	−0.12, 0.08	−.02	<.001

Note

PRDS = Personal Relative Deprivation Scale; Power = Personal Sense of Power Scale; *sr* ^2^ = semi‐partial correlation‐squared.

a *p *<* *.05.

## STUDY 2

Personal relative deprivation, by definition, requires comparison with others (Smith *et al*., 2012). Although the tendency to compare oneself with others is ubiquitous, individual differences exist in people's tendencies to engage in social comparisons (Buunk & Gibbons, 2006), and these individual differences predict people's experiences of PRD (Callan, Kim, & Matthews, 2015b). Further, research has shown that a tendency to make social comparisons is linked positively to materialism (e.g., Chan & Prendergast, 2007; Mandel, Petrova, & Cialdini, 2006; Richins, 1995; Sirgy, 1998). In Studies 2 and 3, then, we examined whether individual differences in the tendency to make social comparisons is one precursor to PRD, which, in turn, relates to materialistic values and goals.

It is likely, however, that not all social comparison tendencies are relevant to either the experience of PRD or materialistic values and pursuits. Specifically, since Festinger's (1954) original formulation of social comparison theory, researchers have distinguished between social comparisons of abilities and social comparisons of opinions (for reviews, see Suls, Martin, & Wheeler, 2000; Wheeler, Martin, & Suls, 1997). The former are typically instigated by self‐evaluative questions of ‘how am I doing’ (Dakin & Arrowood, 1981), whereas the latter concern questions of ‘what shall I believe, like, or feel’ (Suls *et al*., 2000). Given this distinction, a tendency to make opinion comparisons (e.g., ‘My co‐worker feels more strongly about affirmative action than I do’) might be less relevant to people's experiences of PRD, and therefore materialism, than a tendency to make ability comparisons (‘My co‐worker has more than I do’).

Along with assessing PRD and the relative importance of financial success, in Study 2 we employed the Iowa‐Netherlands Comparison Orientation Measure (INCOM; Gibbons & Buunk, 1999) to assess individual differences in both the tendencies to make opinion and ability comparisons. We expected ability comparisons, but not opinion comparisons, to relate to the perceived relative importance of financial success through PRD. The results of an exploratory study we conducted prior to Study 2 lend weight to this hypothesis: Individual differences in ability comparisons, but not opinion comparisons, related to PRD and the perceived importance of financial success.^1^ Full details of this study are available in the Supporting Information.

## Method

### Participants

Participants from the United States were recruited through MTurk (*N *=* *381, 51% male; *M*_age_ = 35.95, *SD*_age_ = 11.29). Twenty‐nine additional participants were excluded because of duplicate IP addresses (*n *=* *11) or failing a basic attention check item (*n *=* *18).

### Procedure and materials

Participants completed the following measures in order:

#### Iowa‐Netherlands Comparison Orientation Measure (INCOM)

We employed the INCOM to measure participants’ tendency to engage in social comparisons (Gibbons & Buunk, 1999). The INCOM is comprised of 11 items that measure people's tendencies to engage in social comparisons of abilities (e.g., ‘I often compare myself with others with respect to what I have accomplished in life’; ‘If I want to find out how well I have done something, I compare what I have done with how others have done’) and opinions (e.g., ‘If I want to learn more about something, I try to find out what others think about it’; ‘I often try to find out what others think who face similar problems as I face’). Individuals scoring higher on the INCOM seek out more comparisons and spend more time engaging in social comparisons (see Buunk & Gibbons, 2006). Higher scores indicate stronger tendency to engage in social comparisons of ability and opinion.

#### PRDS

Participants completed the five‐item PRDS used in Study 1.

#### Relative importance of financial success

We used the Aspiration Index (Kasser & Ryan, 1993, 1996) to assess the perceived importance of financial success relative to other life‐goals. The Aspiration Index contained seven different life‐goal categories (self‐acceptance, affiliation, community feeling, physical fitness, financial success, attractive appearance, and social recognition). Each life‐goal was assessed with four or five items (32 items in total). Participants rated the importance of each statement on a scale ranging from 1 (*not at all important*) to 5 (*very important*).

Following previous research (Dittmar *et al*., 2014), we operationalized materialism in Study 2 as the importance participants placed on financial success as a life‐goal (four items, e.g., ‘You will have a lot of expensive possessions’; ‘You will be financially successful’) relative to the other life‐goals. This was achieved by subtracting each participant's mean across all domains from the mean of the four items assessing their perceived importance of financial success (cf. Sheldon & McGregor, 2000; Sheldon, Sheldon, & Osbaldiston, 2000). This yields a measure of the relative centrality of financial success to each participant's value system (see Kasser & Ryan, 1993, 1996). Higher scores indicate greater perceived importance of financial success relative to other life‐goals.

#### Income and education

Participants reported their annual income and level of educational attainment as per Study 1.

## Results and discussion

Descriptive statistics, alpha reliabilities, and correlations among the measures are shown in Table 3. Personal relative deprivation and the tendency to make ability comparisons correlated significantly with the relative importance of financial success in the expected directions. The tendency to make opinion comparisons did not correlate significantly with the relative importance of financial success. Although opinion comparisons correlated significantly with PRD, a multiple regression analysis with opinion and ability comparisons predicting PRD showed that opinion comparisons did not uniquely predict PRD (*b* = −.05, β = −.03, *SE *= .09), *t*(378) = −.56, *p* = .58, *sr* ^2^ = .0007, over and above the contribution of ability comparisons (*b* = .39, β = .33, *SE* = .07), *t*(378) = 5.87, *p *<* *.001, *sr* ^2^ = .08. Annual household income and educational attainment did not correlate significantly with the relative importance of financial success.

**Table 3:** Descriptive statistics and intercorrelations for measures used in Studies 2 and 3

Measures	Mean (*SD*)	1.	2.	3.	4.	5.	6.
Study 2 (*N *=* *381)							
1. INCOM‐ability	3.14 (.90)	(.87)					
2. INCOM‐opinion	3.70 (.67)	.47^a^	(.75)				
3. PRDS	3.25 (1.08)	.31^a^	.12^a^	(.82)			
4. Financial Success	−.33 (.65)	.27^a^	−.004	.35^a^	–		
5. Income ($)	51.6k (35.5k)	.13^a^	.04	−.25^a^	.03	–	
6. Education	2.80 (.68)	.09	.02	−.01	.04	.34^a^	–
Study 3 (*N *=* *299)							
1. INCOM‐ability	3.37 (.86)	(.87)					
2. PRDS	3.15 (1.01)	.40^a^	(.81)				
3. MVS	3.98 (1.23)	.60^a^	.44^a^	(.90)			
4. Income (£)	32.4k (20.9k)	−.06	−.34^a^	−.09	–		

Note

INCOM = Iowa‐Netherlands Comparison Orientation Measure; PRDS = Personal Relative Deprivation Scale; Financial Success = relative importance of financial success from the Aspiration Index; MVS = Material Values Scale.

When applicable, alpha reliabilities are presented in parentheses along the diagonal.

a *p *<* *.05.

**Figure 1:**
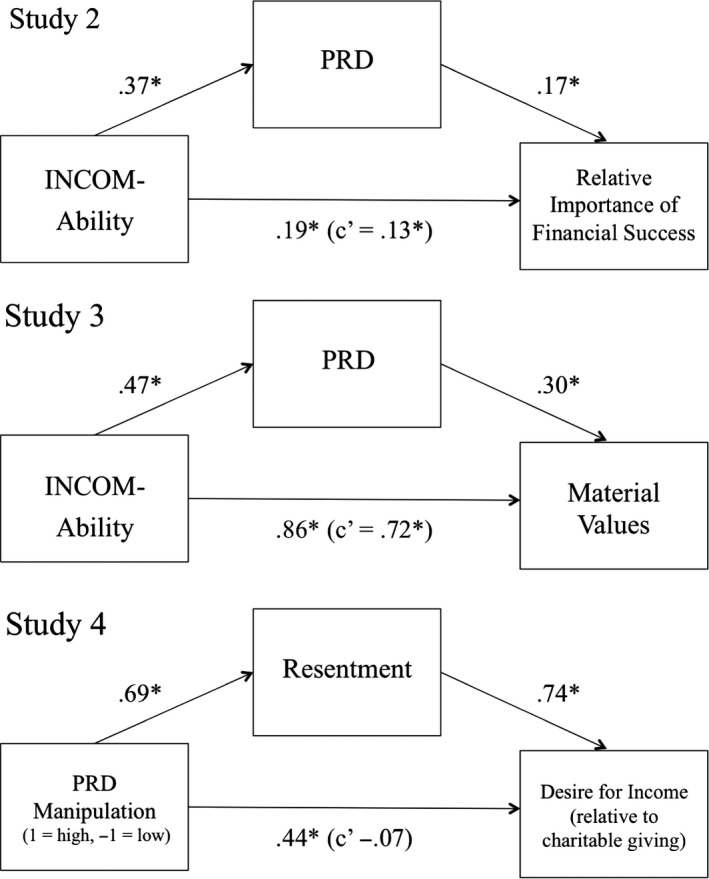
Mediational models for Studies 2, 3, and 4. INCOM = Iowa‐Netherlands Comparison Orientation Measure; PRD = personal relative deprivation. Values depict unstandardized regression coefficients. *p <* *.05.

Using Preacher and Hayes's (2008) bootstrapping procedure, we tested the indirect effect of the tendency to make ability comparisons on the relative importance of financial success through PRD (see Figure 1). The results showed that PRD mediated the relation between social comparison of abilities and the relative importance of financial success (10,000 resamples; indirect effect = .065, 95% bias‐corrected and accelerated confidence interval [BCa CI] of .038 and .101), suggesting that one reason why social comparison of abilities is related to the relative importance of financial success is PRD. The same analyses controlling for annual income and educational attainment revealed similar results (indirect effect = .080, 95% BCa CI of .049 and .120).

## STUDY 3

In Study 3, we aimed to replicate our Study 2 findings using a measure of material values (as per Study 1) among a sample of British participants. Given our Study 2 findings (see also the Supporting Information), we focused on PRD as a mediator of the relation between social comparison of abilities and material values.

## Method

### Participants

Participants were from the United Kingdom (*N *=* *299; 53% male; *M*_age_ = 34.64, *SD*_age_ = 11.61) and completed a brief online survey through either ProlificAcademic.co.uk or CrowdFlower.com (*n*s = 153 and 146, respectively) for a nominal payment. Fourteen additional participants were excluded because of duplicate IP addresses (*n *=* *4) or not being UK residents (*n *=* *10). The latter participants were removed because we asked participants to provide their annual household income in pound sterling.

### Procedure and materials

Participants completed the six‐item social comparison of abilities subscale of the INCOM, the five‐item PRDS, and the nine‐item short form of the MVS (Richins, 2004) in order. Along with reporting their age and gender, participants provided their annual household income using an 18‐point scale with values ranging from 1 (*less than £5,000*) to 18 (*£85,001 and above*), with each option spanning £4,999. None of the measures included an attention check item.

## Results and discussion

Descriptive statistics, alpha reliabilities, and correlations among the measures are shown in Table 3. Social comparison tendency, PRD, and material values all correlated significantly with each other in the expected directions. Annual household income did not correlate significantly with material values. As shown in Figure 1, bootstrapped mediation analyses showed that PRD mediated the relation between social comparison tendency and material values (10,000 resamples; indirect effect = .138, 95% BCa CI of .073 and .217). The same analyses controlling for annual household income revealed nearly identical results (indirect effect = .137, 95% BCa CI of .073 and .217).

## STUDY 4

One limitation of Studies 1 to 3 is the use of cross‐sectional designs. We therefore cannot rule out the possibility that rather than our proposed model (i.e., social comparison → PRD → materialism), materialism might lead to PRD (e.g., through overspending), or that feeling resentful might make people more attuned to what others have. In Study 4, we experimentally manipulated adverse social comparisons by convincing participants that they had either less or the same level of discretionary income compared to people with the same background characteristics (e.g., age, gender, education). This manipulation has been shown to increase resentment and a sense of unfairness (Callan *et al*., 2008) and lower subjective social status (Brown‐Iannuzzi, Lundberg, Kay, & Payne, 2015), even though actual financial position is held constant between experimental conditions. In contrast to other manipulations (e.g., those involving unjust vs. no government decisions within a hypothetical commons dilemma game; Zhang *et al*., 2015), this manipulation specifically varies adverse social comparisons with similar others and therefore is directly tied to the construct of PRD. Following Smith *et al*.'s (2012) model of relative deprivation, we also measured resentment with one's current level of discretionary income as a potential mediator of the effect of the relative discretionary income manipulation on materialism.

## Method

### Participants

Participants were 164 staff and students from the University of Essex who were recruited through a research participant database (76% women; *M*_age_ = 21.74, *SD*_age_ = 3.50). They participated in exchange for course credit or £3. Ten additional participants were not included in analyses because, when probed during debriefing, they were suspicious of the veracity of the feedback they received (*n *=* *3) or did not correctly understand the meaning of the feedback (*n *=* *7).

### Procedure and materials

#### Manipulation of personal relative deprivation

We told participants that the study was part of an ongoing research project examining trends in the discretionary income of students and staff at the University of Essex. We informed them that a computerized survey would provide them with feedback about how their discretionary income compares with the discretionary income of people who matched their ‘personal profile’ in our database, which would be determined by the information they provided.

Ostensibly to create their ‘profile’, participants first completed a scale about their financial beliefs and behaviours (Callan *et al*., 2011) and a personality inventory (Gosling, Rentfrow, & Swann, 2003). Next, they provided background demographic information (i.e., gender, age, employment status, marital status, university major if applicable, years of education, and whether they live at home) and reported their annual household income and average monthly spending over the previous 6 months on housing, utilities, food, clothing, transportation, and debt. Once participants had entered their information, they were shown a screen that read:

On the basis of the information you provided, we will now calculate your Comparative Discretionary Income Index (CDI Index) Score. The CDI index measures a person's standing in terms of his/her average monthly discretionary income relative to the discretionary income of similar others. Based on the information you provided, the index will produce a score using your profile and the information in our StatsPlus™ database from people who match your profile. The score will tell you in pounds (£) how much average monthly discretionary income you have relative to people who match your profile. Depending on current database activities, the process may take up to a minute to complete.

Participants then saw a series of progress screens that were designed to create the impression that their ‘personal profile’ was being constructed and their ‘CDI Index Score’ calculated. Once ‘finished’, participants saw their CDI Index Score, which was presented in large font size within a large black rectangle. Randomly determined, the CDI Index Score that participants were shown was either ‘£ −313’ (*n *=* *80) or ‘£ +54’ (*n *=* *84; the experimenter was blind to the condition). Participants read the following about the meaning of their score:

**How to interpret your StatsPlus™ CDI Index Score**

Your CDI Index Score was derived from statistical analyses using both the information from your profile and the information in our database from people who matched your profile. Your CDI Index Score represents on average how much monthly discretionary income you have relative to people who are highly similar to you in personal characteristics and background. A negative (‐) CDI Index Score means that you have on average less discretionary income than similar others. A positive (+) CDI Index Score means you have on average more discretionary income than similar others.

Participants were given a form to write down their score to ‘make it available to the researcher for use in his/her study (if requested)’.

#### Resentment

After participants wrote down their respective CDI Index Score, they rated how dissatisfied, satisfied (reverse‐scored), and resentful they felt about their current level of discretionary income (1 = *Not at all,* 7 = *Very*). These items were averaged to form a composite measure of resentment (α = .76).

#### Desire for more discretionary income and giving to charity

We designed a measure of the relative importance of financial success that was modelled after the Aspiration Index but tailored towards participants’ current concerns about their level of discretionary income compared to their current desire to give to charity. Across five items, participants indicated how much they desired to increase their discretionary income, how important more discretionary income seemed to them, how much they wanted more discretionary income, how motivated they were to obtain more discretionary income, and how much they felt like they needed more discretionary income (e.g., ‘How important does having more discretionary income seem to you right now?’; 1 = *Not at all*, 7 = *Extremely*; α = .93). Next, they completed five identically phrased items about their current desire to give to charity (e.g., ‘How important does giving money to charity seem to you right now?’; α = .95). We chose these items to represent a goal, akin to ‘community feeling’ from the Aspiration Index, that is opposite to the goal of financial success in Grouzet *et al*.'s (2005) circumplex model of goal contents. Following our approach in Study 2 using the Aspiration Index, and consistent with Dittmar *et al*.'s (2014) definition of materialism noted above, we therefore operationally defined materialism as the perceived importance of attaining more discretionary income relative to giving more to charity, which was calculated by subtracting each participant's mean across the charity items from the mean of the ‘wanting more discretionary’ income items.^2^ Finally, participants were debriefed and probed for their correct understanding of the meaning of their ‘CDI Index Score’.

## Results and discussion

Participants who learned that they had less monthly discretionary income than people like them reported feeling significantly more resentful (*M *=* *4.18, *SD* = 1.39) than did participants who learned that they had roughly the same monthly discretionary income as similar others (*M *=* *2.80, *SD* = 1.28), *t*(162) = 6.63, *p *<* *.001, *d *=* *1.04 (95% CI of the mean difference: 0.97, 1.79). Moreover, participants who learned that they had less monthly discretionary income than similar others reported a greater desire for more discretionary income relative to giving more to charity (*M *=* *1.68, *SD* = 1.94) than those who learned their discretionary was roughly the same as others (*M *=* *.80, *SD* = 2.07), *t*(162) = 2.78, *p *=* *.006, *d *=* *0.43 (95% CI of the mean difference: 0.25, 1.49).

Resentment and the relative desire for more discretionary income were significantly correlated, *r *=* *.54, *p *<* *.001. As shown in Figure 1, bootstrapped mediation analyses showed that resentment mediated the effect of the manipulation on the relative desire for more discretionary income (10,000 resamples; indirect effect = .51, 95% BCa CI of 0.304 and 0.757).

Study 4 revealed that a manipulation of *relative* deprivation increased the relative importance participants placed on acquiring more discretionary income. One potential limitation of these findings is that participants might have reported feeling more resentful and wanting more discretionary income relative to giving more to charity when they learned they had less income than similar others because they believed these responses were what the researchers expected. Although we cannot completely rule out such demand characteristics, it is important to highlight that our manipulation procedure did not inform participants that they did not have less discretionary income in any absolute sense – instead, participants learned that they had less (or roughly the same) level of discretionary income compared to people like them. Our cover story was as much about ‘trends in discretionary income’ as it was about the feedback we gave participants about their ‘profile’, yet ancillary analyses showed that participants’ actual household income did not correlate significantly with how resentful they felt about their current level of discretionary income, *r *=* *−.04, *p *=* *.63. Given our cover story, reporting low income could have plausibly been as much of a demand characteristic for reporting higher resentment as the relative income information we provided, but income did not meaningfully correlate with resentment. Nonetheless, it will be important for future research to assess the effects of such adverse social comparisons using more indirect measures.

Although participants lower in annual household income reported wanting more discretionary income relative to giving to charity, *r *=* *−.18, *p *=* *.02, controlling for household income did not alter the effect of the relative deprivation manipulation on participants’ desire for more discretionary income relative to giving more to charity, *F*(1,161) = 10.79, *p *=* *.001. Thus, the effect we observed here has less to do with beliefs about not having money than beliefs about not having money *relative* to similar others – learning that they had less discretionary income relative to others increased the relative importance participants placed on achieving financial success above and beyond their absolute financial position.

## STUDY 5

Our first four studies found that PRD positively correlated with participants’ self‐rated beliefs, values, or goals associated with acquiring money and possessions. To extend these findings in a new direction, in Study 5 we explored whether PRD might also predict people's *spending preferences*, particularly in terms of how much of a financial windfall they would spend on things they wanted for themselves relative to other types of possible expenditures (e.g., savings, giving to charity). Participants were asked to complete a task where they imagined they were given $20,000 and had to spend it across a number of spending categories, including buying things they wanted or needed. Several studies using this windfall expenditure task have shown that people higher in materialism, as measured by the MVS or Aspiration Index, spend more on things they want than people lower in materialism (Kasser, 2005; Kasser *et al*., 2014; Richins, 2004; Richins & Dawson, 1992); if people higher (vs. lower) in PRD place greater importance on acquiring possessions (i.e., are more materialistic), then they should prioritize buying things they wanted when spending a windfall.

As in our previous studies, we also asked participants to report their annual household income to control for their absolute financial status. Following Mick's (1996) recommendation for research on materialism, we also included a measure of socially desirable responding (SDR) to explore whether the relation between PRD and windfall spending might be confounded by SDR.

## Method

### Participants

Participants were from the United States or Canada (*N *=* *799; 52% male; *M*_age_ = 34.96, *SD*_age_ = 11.56) who completed an online survey through either MTurk (*n *=* *528) or Crowdflower.com (*n *=* *271). Eighty‐five additional participants were excluded because of duplicate IP addresses (*n *=* *26) or failing a basic attention check item (*n *=* *59). Due to a programming error, 60 participants from the United Kingdom were also able to complete the survey; they were not included in analyses because our measures of windfall spending and annual household income asked participants to provide their responses in dollars.

### Procedure and measures

Participants completed these measures in order:

#### Socially desirable responding

We used Stöber's (2001) Social Desirability Scale‐17 (SDS‐17) to measure people's tendency to present themselves favourably (e.g., ‘I sometimes litter’; ‘I always eat a healthy diet’). Participants answered ‘True’ or ‘False’ to the statements; participants higher in socially desirable responding (SDR) are expected to over‐report ‘good’ behaviour and under‐report undesirable behaviour (Mick, 1996). The SDS‐17 has good internal reliability and convergent and discriminant validity (Blake, Valdiserri, Neuendorf, & Nemeth, 2006; Stöber, 2001).

#### Personal relative deprivation

Participants completed the five‐item PRDS used in Study 1.

#### Imaginary windfall expenditure

To examine participants’ spending preferences, we asked them to imagine they had unexpectedly received $20,000 and could spend it in seven ways: ‘Buy things I want or need’, ‘Give to charity or church organizations’, ‘Give or lend to friends or relatives’, ‘Travel’, ‘Pay off debts’, ‘Savings or investments’, and ‘Other’ (Richins & Dawson, 1992). Participants provided only dollar amounts for each category (i.e., no itemized textual responses by category were solicited). Participants were free to divide the money between the categories as they wished, but had to ensure the total spending summed to $20,000. The online survey showed participants a running total of their ‘spending’, and they could not advance to the next page unless the total value equalled $20,000.

#### Income

Participants reported their annual household income before taxes as per Study 1. Participants also reported their gender and age.

## Results and discussion

Descriptive statistics and correlations among the measures are shown in Table 4. Personal relative deprivation and SDR were significantly negatively correlated, such that participants higher in SDR reported lower PRD, suggesting the possibility that participants under‐report their perceived relative deprivation. In terms of the imaginary windfall spending, PRD correlated significantly with the amount of money participants spent on things they wanted or needed, such that participants higher in PRD tended to spend more on things they wanted or needed relative to the other spending categories.

**Table 4:** Descriptive statistics and correlations among measures (top section) and estimates from Tobit and OLS regression models predicting the amount of money participants spent on ‘buy things I want or need’ from PRDS, SDR, and annual household income (bottom section) in Study 5

Measures	Mean (*SD*)	PRD	SDR	Income
1. PRD	3.12 (1.02)	(.83)		
2. SDR	8.57 (3.64)	−.181^a^	(.77)	
3. Income	$52,933 (38,430)	−.253^a^	−.054	–
4. Buy things I want or need	$3,829 (3,898)	.158^a^	−.081^a^	−.102^a^
5. Give to church or charity	$639 (1,156)	−.118^a^	.137^a^	.002
6. Give or lend to friends or relatives	$903 (1,609)	−.016	−.013	−.051
7. Travel	$1,763 (2,271)	−.013	−.010	.032
8. Pay off debts	$5,261 (5,357)	−.034	.021	.018
9. Savings or investments	$6,969 (5,412)	−.065	.008	.086^a^
10. Other	$636 (2,039)	.053	.024	−.079^a^

	Robust Tobit model			OLS model			
	Estimate	*SE*	*p*‐value	*b* [95% CI]	*SE*	*p*‐value	*sr* ^2^
PRD	485.45	159.77	.002	489.52 [214, 765]	140.41	<.001	.014
SDR	−87.15	44.00	.048	−66.24 [−141, 9]	38.23	.084	.004
Income	−0.01	0.01	.017	−0.007 [−.015, −.002]	0.004	.044	.005

Note

PRD = personal relative deprivation; PRDS = Personal Relative Deprivation Scale; SDR = socially desirable responding; OLS = ordinary least squares. When applicable, alpha reliabilities are presented in parentheses along the diagonal.

a *p *<* *.05.

As depicted in Table 4, a multiple regression analysis showed that PRD remained a significant predictor of the amount participants reported they would spend on things they wanted or needed while controlling for SDR and annual household income. The windfall expenditure data were highly positively skewed and contained a large number of zero responses, indicative of censoring (participants cannot indicate a negative allocation of funds to a given category). Such data cannot be symmetrized by log transformation, so we complemented our ordinal least squares (OLS) regression with a robust Tobit model, a standard approach to analysing censored econometric data (Wooldridge, 2010). This was performed using the *zelig* package (Imai, King, & Lau, 2007, 2008) in R. As shown in Table 4, the Tobit model confirmed that PRD significantly predicted the amount participants spent on things they wanted or needed over and above the contributions of SDR and annual household income. Tobit regressions controlling for SDR and annual household income were also run for each of the other spending categories of the windfall expenditure task. These analyses revealed that PRD uniquely predicted the amount of money participants were willing to donate to charity (*B *=* *−256.4, *p *=* *.002) but no other spending category of the windfall expenditure task (all *p*s > .125). Thus, over and above SDR and annual household income, higher PRD was associated with spending more on the self and giving less to charity.

## GENERAL DISCUSSION

In five studies, we found that PRD was associated with materialistic values and goals. The relation between PRD and materialism was not confounded by other individual difference factors known to influence materialism (i.e., personal sense of power, self‐esteem, emotional uncertainty, and socioeconomic status, SDR; Studies 1 and 5), and the tendency to make social comparisons of abilities (but not opinion comparisons) correlated positively with PRD, which, in turn, related to materialistic values and goals (Studies 2 and 3). Although previous studies have linked a general tendency to make social comparisons to higher materialism (e.g., Chan & Prendergast, 2007; Mandel *et al*., 2006; Richins, 1995), none have shown that this relationship specifically occurs only for a tendency to make ability comparisons. In Study 4, we provided causal evidence that unfavourable social comparisons increase the relative importance participants placed on achieving financial success. In Study 5, we found that participants higher in PRD planned to spend more on things they wanted relative to other spending categories than participants lower in PRD. Thus, the role the PRD plays in materialism is evident not only in people's self‐rated beliefs, values, and goals but also in their spending preferences.

Taken together, our findings suggest that PRD contributes to materialism. It is important to highlight that these studies are the first to show that *absolute* deprivation, which we measured via annual household income, did not account for the relation between PRD and materialism across our studies, and Study 4 showed that randomly assigning participants to learn that they had less (vs. similar) discretionary income than people like them increased the relative importance they placed on acquiring money. Thus, simply lacking financial resources was, by and large, not associated with the adoption of materialistic values and goals across our studies – instead, materialism was predicted by participants’ concerns about their *relative* deprivation.

One alternative possibility, however, is that the relation between PRD and materialism might be moderated by absolute income, such that the associations and effects we have observed might occur only – or more strongly – for people experiencing absolute financial deprivation. Moderated regression analyses of our data suggest this is not the case: Annual household income did not significantly modulate the relation between PRD and (1) scores on the MVS (Studies 1 and 3 collated, *N *=* *692; *p *=* *.19), (2) the relative importance participants placed on financial success (combination of Study 2 and Study S1 in the Supporting Information, *N *=* *740; *p *=* *.99), and (3) windfall spending on things participants wanted or needed (Study 5, *p *=* *.89). Furthermore, the effect of the manipulation of adverse social comparisons on the relative desire for more discretionary income in Study 4 was not significantly modulated by annual household income (*p *=* *.09; if anything, the effect was slightly stronger for participants higher in income). Thus, as we noted in the Introduction, even people who are objectively ‘wealthy’ can feel resentful about what they have compared with what others like them have, and our findings suggest that these feelings correlate with the importance they place on acquiring money and possessions.

Although we found that our results were not due to or moderated by absolute household income, it will be important for future research to probe the roles that other economic indicators of people's spending power might play in the relation between PRD and materialism. For example, the relation between PRD and the adoption of materialistic goals might depend on one's existing possessions and assets, how much people believe they are able to spend money on the things they want, or even one's access to credit (see Matthews, Gheorghiu, & Callan, 2016, for a discussion of this issue).

The results of our studies contribute to a growing body of research highlighting that PRD is a distinct and important predictor of a variety of internal states and individual behaviours (e.g., Callan, Kim, Gheorghiu, & Matthews, 2016; Callan *et al*., 2015b; Mishra & Novakowski, 2016; Smith & Pettigrew, 2015; Smith *et al*., 2012; Tabri, Dupuis, Kim, & Wohl, 2015). Insofar as materialism has detrimental consequences for individuals and for society, interventions aimed at reducing it might focus on ameliorating people's resentment and sense of unfairness stemming from their *relative* material standing. Moreover, increases in materialism may have less to do with increasing income inequalities at the societal level than potentially greater access to unfavourable social comparisons at the interpersonal level (e.g., through social media; de Vries & Kühne, 2015).

Working from Dittmar *et al*.'s (2014) definition of materialism, we focused on individual differences in the values and importance people placed on attaining money and possessions. One avenue for future research will be to probe whether social comparison tendencies and PRD further relate to the known behavioural consequences of materialism, such as increased debt and actual spending behaviours (Garðarsdóttir & Dittmar, 2012). An additional possibility open for future enquiry is the idea that the causal relation between PRD and materialism is bidirectional, such that over time higher PRD might affect increased spending which, in turn, leads to increased PRD, such as through the accumulation of debt or shifts in social comparison targets – potentially creating a pernicious ‘feedback loop’.

## Funding

This work was supported by grant RPG‐2013‐148 from the Leverhulme Trust and studentship ES/J500045/1 from the Economic and Social Research Council.

## Supporting information


**Appendix S1.** Supplementary content.

## Footnotes

1 Following Gibbons and Buunk's (1999) suggested use of the INCOM, our original analysis strategy for this exploratory study was to use the full measure (i.e., averaged across ability and opinion comparisons) to predict personal relative deprivation and the importance of financial success. Recent research, however, suggests that the two‐factor solution – with ability and opinion comparisons as separate but correlated factors – better fits the data and is more valid (Callan *et al*., 2015b; Schneider & Schupp, 2014). Therefore, the current Study 2 is a confirmatory study of an exploratory study using the separate subscales as predictors.

2 This exact measure and the relative importance of financial success measure from the Aspiration Index were significantly correlated, *r* = .45, *p* < .001, in a separate validation study with participants recruited through MTurk (*N* = 89; *M*_age_ = 37.29; *SD*_age_ = 12.22; 56% male).
