# Supplementary material for: Social comparison, personal relative deprivation, and materialism
Source: Br J Soc Psychol. 2016 Nov 23;56(2):373–92. doi: 10.1111/bjso.12176 (PMC5484278; doi:10.1111/bjso.12176)
Supplement: Supplementary file 1 — Appendix S1. Supplementary content. [file BJSO-56-373-s001.docx]

**Supplementary Content**

Here we report the methods and results of an additional study exploring the relations among social comparison tendencies, personal relative deprivation, and the relative importance of financial success.

**Method**

**Participants**

Participants from the USA (N = 359; 51% male; *M_age_* = 35.35, *SD_age_* = 12.21) completed an online survey through MTurk or CrowdFlower.com for a nominal payment (*n*s = 182 and 177, respectively). Seventeen additional participants were excluded because of duplicate IP addresses (*n* = 2) or failing a basic attention check item (*n* = 15).

**Procedure and Materials**

The procedure and materials used followed exactly those used in Study 2 of the main text. Specifically, participants completed the 11-item Iowa Netherlands Comparison Orientation Measure (INCOM), the Personal Relative Deprivation Scale (PRDS), and the Aspiration Index. They also reported their annual household income and educational attainment.

**Results and Discussion**

**Confirmatory Factor Analysis**

Following Gibbons and Buunk’s (1999) recommended use of the INCOM, our *a priori* analysis strategy was to examine the relations among social comparison tendency, personal relative deprivation, and the relative importance of financial success using the *full* INCOM (i.e., assuming a single factor model). However, consistent with recent findings (Callan, Kim, & Matthews, 2015b; Schneider & Schupp, 2014), a confirmatory factor analysis showed that the one-factor solution for the INCOM provided worse fit (*χ*^2^ = 400.85, df = 43, *p* < .001; CFI = .783; TLI = .723; RMSEA = 0.152; SRMR = .100) than the two-factor solution (i.e., with the ability and opinion subscales as distinct but correlated factors; *χ*^2^ = 139.36, df = 42, *p* < .001; CFI = .941; TLI = .923; RMSEA = .080; SRMR = .074).^[[1]](#footnote-1)^ Therefore, below we report the results from the full INCOM as planned, but we also report exploratory analyses for the ability and opinion comparison subscales separately.

**Correlation and Mediation Analyses**

Descriptive statistics and correlations among the measures are shown in Table S1. Overall social comparison tendency, personal relative deprivation, and the relative importance of financial success all correlated significantly with each other in the expected directions. Annual household income and educational attainment did not correlate significantly with the relative importance of financial success. Ability comparisons, but not opinion comparisons, correlated significantly with personal relative deprivation and the relative importance of financial success.

Using Preacher and Hayes's (2008) bootstrapping procedure for testing indirect effects, we tested the indirect effect of social comparison of abilities on the relative importance of financial success through personal relative deprivation (see Figure S1). The results showed that personal relative deprivation mediated the relation between ability comparisons and relative importance of financial success (10,000 resamples; indirect effect = .044, 95% bias-corrected and accelerated confidence interval [BCa CI] of .018 and .080). The same analyses controlling for annual income and educational attainment revealed virtually identical results (indirect effect = .048, 95% BCa CI of .020 and .084).

**TableS1.** *Descriptive Statistics and Intercorrelations (N = 359)*

| Measures | Mean (*SD*) | 1. | 1a. | 1b. | 2. | 3. | 4. | 5. |
| --- | --- | --- | --- | --- | --- | --- | --- | --- |
| 1. INCOM | 3.35 (.71) | (.87) |  |  |  |  |  |  |
| 1a. ability | 3.14 (.87) | .93* | (.87) |  |  |  |  |  |
| 1b. opinion | 3.60 (.72) | .83* | .56* | (.77) |  |  |  |  |
| 2. PRDS | 3.18 (1.00) | .21* | .31* | .01 | (.81) |  |  |  |
| 3. Financial Success | -.38 (.65) | .19* | .27* | .03 | .26* | -- |  |  |
| 4. Income ($) | 49.74K (34.8K) | .06 | .05 | .06 | -.23* | -.004 | -- |  |
| 5. Education | 2.80 (.68) | .09 | .06 | .10 | -.12* | -.03 | .28* | -- |

*Note.* INCOM = Iowa-Netherlands Comparison Orientation Measure. PRDS = Personal Relative Deprivation. Financial Success = Relative importance of financial success from the Aspiration Index. When applicable, alpha reliabilities are presented in parentheses along the diagonal.

* *p* < .05.


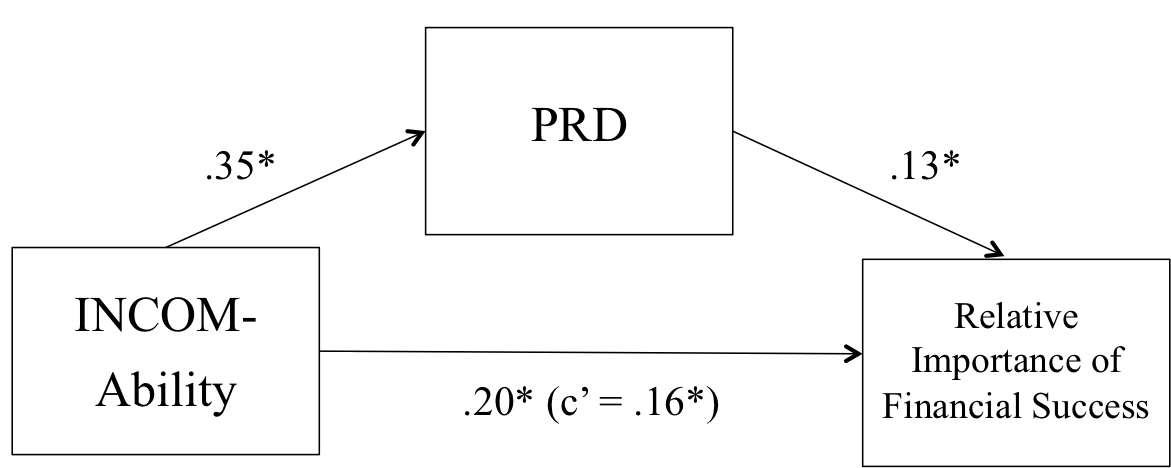


**Figure S1.** Mediational models for supplementary study. INCOM = Iowa-Netherlands Comparison Orientation Measure. PRD = Personal Relative Deprivation. Values depict unstandardized regression coefficients. * *p* < .05.

1. For these analyses, correlations between the error variances for the two reversed worded items were specified (cf. Schneider & Schupp, 2014). [↑](#footnote-ref-1)
